# Reaching beyond family history as inclusion criteria for pancreatic cancer surveillance in high-risk populations

**DOI:** 10.18632/genesandcancer.223

**Published:** 2022-08-29

**Authors:** Louise Wang, Susan M. Domchek, Michael L. Kochman, Bryson W. Katona

**Keywords:** pancreatic cancer surveillance, pancreatic cancer risk, endoscopic ultrasound, MRI

Pancreatic ductal adenocarcinoma (PDAC) is predicted to be the second most deadly cancer in the United States by 2030 [1]. Although stage 1A PDAC 5-year survival is now greater than 80%, the majority of PDAC is diagnosed at more advanced stages, with 5-year survival being less than 10% for stage III/IV disease [2]. The majority of PDAC is sporadic, however up to 10% of PDAC is considered to be familial [3], including individuals with a pathogenic or likely pathogenic variant (PV) in a known PDAC susceptibility gene and/or familial pancreatic cancer, defined as a family with at least two relatives with PDAC who are directly related to one another without known genetic susceptibility.

As surgical resection of early-stage disease offers the highest chance of long-term survival, effective pancreatic cancer surveillance in high-risk individuals (HRIs) is imperative to allow for early detection. While some reports have not shown strong evidence that pancreatic cancer surveillance is effective [4, 5], older [6] as well as more recent data [7] from the Cancer of the Pancreas Screening (CAPS) studies showed that only 5% of surveillance detected PDACs in HRIs were stage IV, while 86% of PDACs diagnosed outside of surveillance were stage IV. Furthermore, 58% of surveillance detected PDACs were stage I, and 5-year survival amongst surveillance detected PDACs was 73% (median overall survival of 9.8 years vs. 1.5 years among HRIs with PDACs detected within and outside of surveillance, respectively) [7]. Notably though, only 26 (1.5%) PDAC cases were diagnosed amongst 1,731 patients enrolled in the CAPS studies [7]. Nonetheless, this data illustrates that PDAC surveillance of HRIs may lead to diagnosis of PDAC at earlier stages with improved long-term survival.

PDAC surveillance guidelines for HRIs carrying a PV in a PDAC risk gene such as *BRCA1*, *BRCA2*, *PALB2*, *ATM*, and genes associated with Lynch syndrome have classically required a family history of PDAC in a first or second degree relative to qualify for surveillance [8]. This contrasts with carriers of higher risk PVs in genes such as *CDKN2A* and *STK11* (lifetime PDAC risk > 15% [8]), where a family history of PDAC is not required for surveillance eligibility. For carriers of a *BRCA1*, *BRCA2*, *PALB2*, *ATM*, or Lynch syndrome PV, restricting PDAC surveillance to those with a family history of PDAC has limitations. First, the majority of these PV carriers who develop PDAC do not have a family history of PDAC [9], and family history of cancer for these PV carriers may also be unknown or uncertain. Furthermore, these individuals could be from small families or have relatives who died from other cancers at a young age, thus reducing the number of at-risk relatives.

To address PDAC surveillance of *BRCA1, BRCA2, PALB2,* and *ATM* PV carriers without a family history of PDAC, our institution started a prospective PDAC surveillance study of these individuals in 2015 (NCT02478892). We recently reported our initial results from 64 PV carriers who underwent at least one surveillance endoscopic ultrasound (EUS) at our institution [10]. Our cohort was predominantly female (72%) and *BRCA2* PV carriers (73%); of these individuals, 44% had a pancreatic abnormality identified on EUS, including 27% with a pancreatic cyst, which are rates similar to those observed amongst HRIs with a family history of PDAC [4, 5]. Eight percent of individuals developed pancreatic cysts on subsequent surveillance exams, and 3% (two individuals) developed PDAC, one of which was detected at stage I.

Given the improved stage shift to local, resectable cancers among HRI undergoing surveillance, the American Society for Gastrointestinal Endoscopy (ASGE) recently released new pancreatic surveillance guidelines recommending consideration of PDAC surveillance for all *BRCA1, BRCA2* and *PALB2* carriers age 50 or older, irrespective of family history [11]. Of note, the NCCN currently only recommends PDAC surveillance for such PV carriers with a first or second degree relative with PDAC [8]. The removal of the family history requirement by the ASGE is a fundamental shift in surveillance recommendations for these PV carriers, expanding surveillance eligibility to an increased number of HRIs. Some centers have begun to offer such surveillance. At our center, we discuss the risks, benefits and uncertainties of surveillance, as well as our preference that if patients decide to undertake such surveillance, they do so as part of a clinical study.

**Figure 1:**
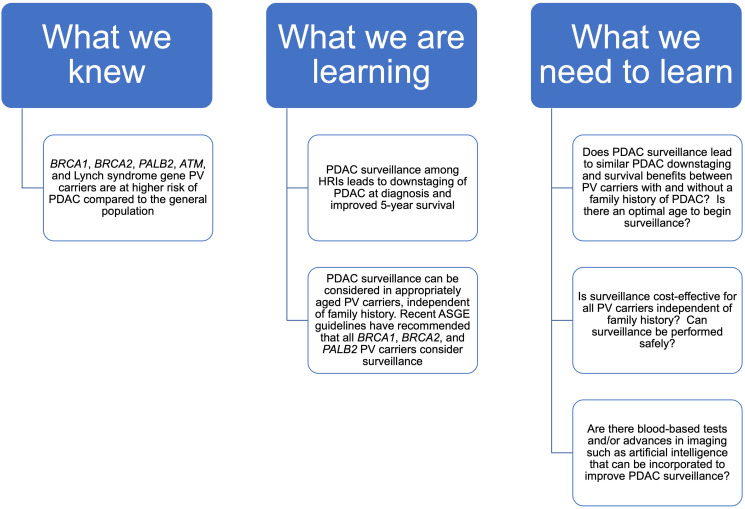
Pancreatic surveillance in PV carriers without a family history of PDAC.

However, there remain multiple critical questions in the field that need further research (Figure 1), including prospective studies evaluating whether surveillance among PV carriers without a family history can downstage PDAC diagnosis and extend PDAC survival similar to higher-risk populations. This will require large cohorts given the elevated, but relatively low, risk of PDAC in PV carriers (amongst all PV carriers, absolute risk by age 80 for PDAC in males and females is 2.9% and 2.3% respectively for *BRCA1* carriers, and 3.0% and 2.3% respectively for *BRCA2* carriers [12], with other studies showing higher risks for *BRCA2* carriers [13]). Furthermore, the optimal age to initiate surveillance (i.e., is age 50 too early for PV carriers without a family history?) as well as whether surveillance should be offered in a gene-specific manner (i.e., to all *BRCA2* carriers but only *BRCA1* carriers with a family history of PDAC?) need further clarification. While we previously found that endoscopic ultrasound is cost effective in individuals with long life expectancy and increased life-time PDAC risk (>10.8%), this risk is higher than expected for a PV carrier without a family history of PDAC [14]. Notably, recent data from the CAPS studies showed only one PDAC was diagnosed per year for every 194 individuals screened [7]. Therefore, cost-effectiveness analyses should be updated to reflect the recent data for PV carriers without a family history and model other inputs, such as age of initial screening and differences in screening modalities such as MRI or EUS.

Additional work will also need to study factors associated with adherence to surveillance intervals, as well as the adverse events and risks that result from surveillance exams including procedural related complications, unnecessary surgeries for low-risk lesions that are discovered on surveillance (5 individuals [0.3%] underwent surgical resection of a low-risk lesion amongst the 1461 subjects in the CAPS5 study [7]), and psychological and/or physical harm that could result from discovery and work-up of incidental lesions identified outside the pancreas. Finally, developing blood-based tests that can be utilized either independently or in conjunction with imaging for PDAC surveillance, as well as improved sensitivity and specificity of imaging modalities, potentially through using artificial intelligence/machine learning, are other important areas where research is needed.

The data supporting PDAC surveillance in HRIs is encouraging, however there remain fundamental uncertainties in the field which will only be resolved by continued close follow-up of HRIs undergoing PDAC surveillance and capturing of this critically important data in clinical studies.
